# Transmission Dynamics of Punique Virus in Tunisia

**DOI:** 10.3390/v14050904

**Published:** 2022-04-26

**Authors:** Khalil Dachraoui, Ifhem Chelbi, Mourad Ben Said, Raja Ben Osman, Saifedine Cherni, Rémi Charrel, Elyes Zhioua

**Affiliations:** 1 Pasteur Institute of Tunis, Unit of Vector Ecology, Tunis 1002, Tunisia; khalil.dachraoui@yahoo.com (K.D.); ifhemc2001@yahoo.fr (I.C.); saifcherni@yahoo.com (S.C.); 2 Higher Institute of Biotechnology of Sidi Thabet, University of Manouba, Manouba 2010, Tunisia; bensaidmourad83@yahoo.fr; 3 Laboratory of Microbiology, National School of Veterinary Medicine of Sid Thabet, University of Manouba, Manouba 2010, Tunisia; 4 National Drug Control Laboratory, Vaccine Control Unit, Tunis 1002, Tunisia; raja.benosman@yahoo.com; 5 Unité des Virus Emergents, Aix-Marseille University, IRD 190, INSERM 1207, 13005 Marseille, France; remi.charrel@univ-amu.fr

**Keywords:** sandflies, phlebovirus, Punique virus, transmission dynamics

## Abstract

A novel phlebovirus, Punique virus (PUNV), was discovered and isolated in 2008 from sandflies from Northern Tunisia. PUNV is now classified as a unique member of the Punique phlebovirus species within the Phlebovirus genus in the Phenuiviridae family (order bunyavirales). In this study, we aimed to investigate the transmission dynamics of PUNV in Tunisia. Sandflies were collected during two consecutive years, 2009 and 2010, by CDC light traps. In 2009, a total of 873 sandflies were collected and identified to the species level. Phlebotomus perniciosus was the most abundant species. One pool of P. perniciosus females collected in autumn contained PUNV RNA, yielding an infection rate of 0.11%. The population densities of circulating sandfly species were assessed during May–November 2010 in Northern Tunisia by using sticky traps. Phlebotomus (Larroussius) perniciosus (71.74%) was the most abundant species, followed by Phlebotumus (Larroussius) longicuspis (17.47%), and Phlebotumus (Larroussius) perfiliewi (8.82%). The densities of dominant sandfly species were found to peak in early spring and again in the autumn. In 2010, species identification was not performed, and sandflies were only discriminated on the basis of sex and collection date. Out of 249 pools, three contained PUNV RNA. Each positive pool allowed virus isolation. The three pools of female sandflies containing PUNV RNA were collected in autumn with an infection rate of 0.05%. These findings provide further evidence that P. perniciosus is the main vector of PUNV in Tunisia, and this phlebovirus is endemic in Tunisia. Our findings provided strong evidence of intensive circulation of PUNV in sandflies and hosts through a viral infection buildup process between sandfly vectors and hosts starting at the beginning of the activity of sandflies in spring to reach a maximum during the second main peak in autumn.

## 1. Introduction

In the Mediterranean basin, sandflies are the main vectors of arboviruses belonging to the Phlebovirus genus, (order Bunyavirales, family Phenuiviridae) affecting human populations where 250 million are at risk [1]. While Toscana virus (TOSV), Adria virus (ADRV), and sandfly fever Turkey virus (SFTV) can cause neuro-invasive infections in humans during the warm season [2,3,4,5,6], other phleboviruses, including sandfly fever Naples virus (SFNV), sandfly fever Sicilian virus (SFSV), and sandfly fever Cyprus virus (SFCV), are the etiological agents of the typical “3-day fever” or “papataci fever” [7,8].

While several sandfly-borne phleboviruses have been detected and/or isolated from sandflies collected from Northern Mediterranean countries, such as Massilia virus (MASV) in France and Portugal [9,10], Granada virus (GRV) in Spain [11], ADRV in Albania [12], and Olbia virus (OLV), Provencia virus (PrV), and Fermo virus (FERV) in Italy [13,14] that are members of SFNV and closely related but distinct from TOSV, only limited data are available from North African and the Middle East region. SFSV was detected in sandflies from Algeria [15,16]. TOSV was detected and/or isolated in sandflies from Morocco and Algeria [17,18]. This is with the exception of Tunisia where several previously known, as well as novel, phleboviruses were detected and/or isolated during the last decade, including TOSV [19], SFSV [20], Utique virus (UTIV), Punique virus (PUNV) [21], Medjerda Valley virus (MVV) [22], and Saddaguia virus (SADV) [20,23]. Taking into account that PUNV was the first phlebovirus to be isolated from sandflies in Tunisia [21], we aimed to study its transmission dynamics in sandfly populations.

## 2. Material and Methods

### 2.1. Collection of Sandflies

Sandflies were collected from the site of Utique (37°08′ N, 7°74′ E), a well-known site of zoonotic visceral leishmaniasis in the north of Tunisia [24], by using CDC miniature light traps (John W. Hock Company, Gainesville, FL, USA). These traps were placed inside houses and in animal shelters located in peri-domestic areas. Traps were carried out from dusk to dawn during the summer of 2009 and 2010. Every year, two campaigns were performed: the first one at the start of the season (May–June), corresponding to the first peak of sandfly activity; the second one in September–October, corresponding to the second larger peak of activity [25]. Traps were collected the following morning and brought back to the laboratory. Each morning, sandflies were collected and pooled with a maximum of 30 individuals per pool.

Collected sandflies, in 2009, were dissected under a cold stereomicroscope on ice to remove the genitalia for species identification, and the rest of the body was placed in a 1.5 mL microfuge tube to be examined for the presence of phlebovirus. Sandflies were identified at the species level by using the identification keys of Croset et al. (1978) [26] with special attention to the atypical form of female *P. perniciosus* that could be confused with *P. longicuspis* [27,28]. Sandflies were pooled with a maximum of 30 individuals per pool according to trapping date, species, and sex, and stored at −80 °C until use.

Because identification of sandflies individually is extremely time consuming, collected sandflies in 2010 were not identified morphologically, but only discriminated on the basis of sex and collecting date. This approach was decided to allow massive collection and promote presentation of the virus for cell culture isolation. However, in term of public health, it is of major importance to point out that this approach induced a potential gap in information due to the inability to morphologically identify individually all collected sandflies in 2010.

Concomitantly to virus detection and isolation, the phenology of sandfly species was studied in the same site. Sticky traps made of paper (20 cm × 20 cm) soaked in castor oil were used to collect sandflies. Thirteen sticky traps (total surface is 1 m^2^) attached with one cord and spaced evenly were suspended 2 m above the ground in one sheep pen. One trapping unit (13 sticky papers) was placed every week for two consecutive nights from May until November 2010. Sandflies were removed from the sticky traps the following day with a fine-haired brush and placed in 95% ethanol and later mounted on glass slides in Mark André medium and identified to species using the identification keys of Croset et al. (1978) [26]. The density of sandflies was recorded as the number of a sandfly species per m^2^ of sticky trap [25].

### 2.2. Virus Detection, Isolation, Sequencing, and Phylogenetic Analysis

Pools of entire sandflies were processed for viral detection and viral isolation as previously described [20,21]. The infection rate of sandflies by phlebovirus (IR) is defined as the number of positive pools divided by the total number of tested specimens × 100 [21].

Viral RNA obtained from infected Vero cells was used to extend sequences obtained at the detection stage. PCR products, obtained using primers located in the polymerase gene (L RNA segment), were cloned and sequenced, giving partial sequences of 201 bp [29]. The obtained sequences were used for genetic and phylogenetic analyses, with homologous sequences of genetically related phleboviruses retrieved from GenBank. Distances and groupings were determined by the pairwise-distance algorithm and neighbor-joining method within MEGA5, and the robustness of the groups were tested using 1000 bootstrap pseudo-replicates.

## 3. Results

### 3.1. Sandfly Trapping and Virus Detection

A total of 873 sandflies were captured in 2009 (461 females and 412 males) (Table 1). *Phlebotomus perniciosus* was the most abundant species (85.91%), followed by *Phlebotomus longicuspis* (11.45%), *Phlebotomus perfiliewi* (1.14%), *Phlebotomus papatasi* (1.14%), and *Sergentomya minuta parotti* (0.34%) (Table 1).

**Figure 1:**
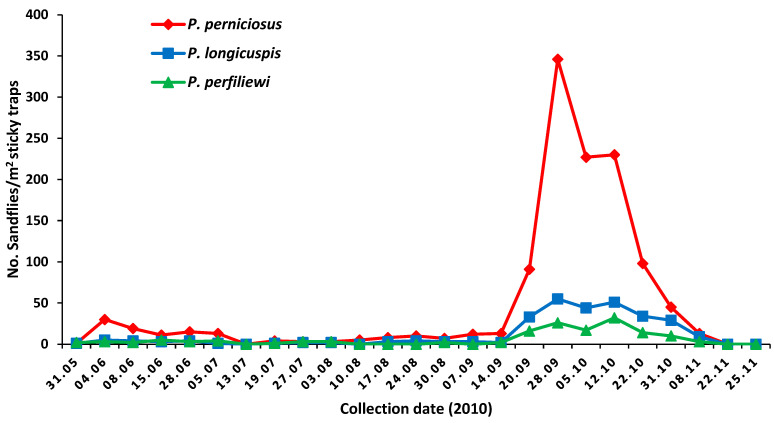
Phenology of sandflies collected in the site of Utique, 2010.

In 2010, the phenology of sandflies in the site of Utique showed that *P. perniciosus* (71.74%) was the most abundant sandfly species, followed by *P. longicuspis* (17.47%), and *P. perfiliewi* (8.82%). Other sandfly species are much less abundant, such as *P. papatasi*, *P. sergenti*, *S. minuta parotti*, *S. christophersi*, and *S. antennata*. The phenology of sandflies of the subgenus *Larroussius,* including *P. perniciosus*, *P. longicuspis*, and *P. perfiliewi,* showed two main peaks: one small on in June and a second larger one in September–October (Figure 1).

A total of 57 pools were formed from collected sandflies in 2009 and used for virus detection. One pool (T101), corresponding to 30 *P. perniciosus* females trapped in October 2009, was found to contain PUNV RNA (Table 2). Thus, the IR of sandflies with PUNV was 0.11%.

Compared with the protocol used in 2009, sandflies that were tested for viruses were not morphologically identified to the species level, but only sorted based on sex and trapping night. This approach was less time consuming and allowed for the process of sandflies with reduced handling time, which is a critical point for preserving infectivity and for increasing the chance of virus isolation. A total of 5,288 sandflies (3547 females and 1740 males) were captured from June to October 2010. Out of 249 pools tested, pools T114, T122, and T192 were positive with at least one of the PCR assay. The IR observed in 2010 was 0.05%. PCR products of the expected size were gel-purified for Sanger sequencing. Pools T114 (30 females collected on 29 August 2010), T122 (30 females collected on 6 September 2010), and T192 (17 females collected on 11 October 2010) contained PUNV RNA.

### 3.2. Virus Isolation and Phylogenetic Study

**Figure 2:**
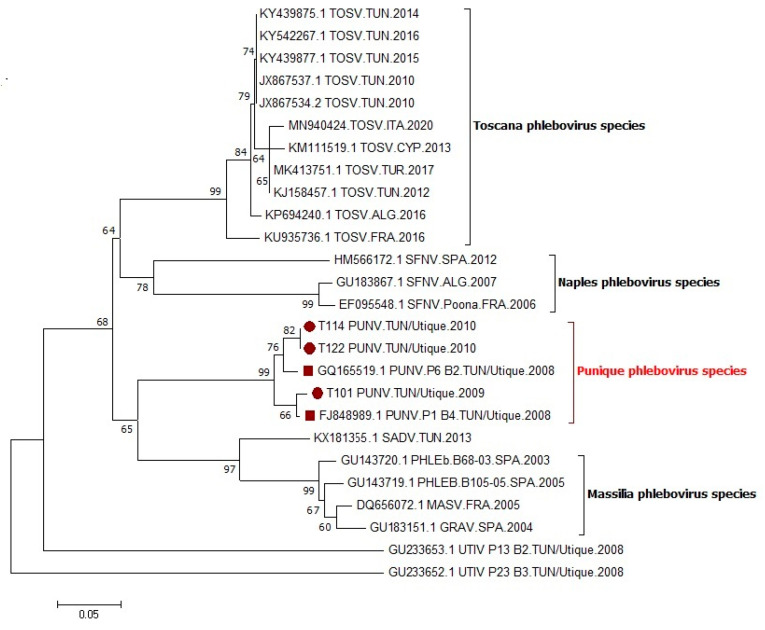
Phylogenetic analysis of PUNV with selected phleboviruses using partial sequences of the polymerase gene available in GenBank. Tree topology was determined by neighbor-joining method within MEGA5, and supported by 1000 bootstrap replicates.

Vero cells inoculated with the T101 (2009), T114 (2010), T122 (2010), and T192 (2010) pools showed a CPE on day seven, confirmed during two additional passages. It is important to point out that T192 produced a CPE, but only the SFN II PCR was positive whereas the NPhlebo I/II system remained negative; since SFN I/II assay targets the nucleocapsid protein gene, the resulting (short) sequence could not be included in the nucleotide alignment produced with partial polymerase gene sequences derived from the NPhlebo PCR products. The phylogenetic tree produced using partial nucleotide sequences in the polymerase gene showed that sequences corresponding to the three strains, T101 (2009), T114 (2010), and T122 (2010) (GenBank accession numbers OM362898, OM362899, OM362900), were most closely related with the sequence of the PUNV strain isolated in 2008, and formed together a monophyletic group (99% bootstrap value) (Figure 2).

## 4. Discussion

Since PUNV was the first phlebovirus detected and isolated from Tunisia, we investigated the circulation of this sandfly-borne virus in sandflies over two consecutive years for a better understanding of its transmission dynamics. During the entomological investigation carried out during two consecutive years, 2009 and 2010, sandflies belonging to the subgenus *Larroussius* were predominantly *P. perniciosus*, *P. longicuspis*, and *P. perfiliewi*. These findings are concordant with previous studies performed in Northern Tunisia showing the predominance of these sandfly species [21,24,30]. The phenology of sandfly species performed from May to November 2010 showed two main peaks: a small one in June and a second larger one in September–October. A similar pattern was also shown in Central Tunisia [25].

In our study, the infection rates of sandflies with PUNV were 0.11% in 2009 and 0.05% in 2010. A similar IR of 0.13% was observed in sandflies collected from the site of Utique in 2008 [21]. Similar results have been reported concerning the infection of sandflies by other phleboviruses in Tunisia. Infection rates of sandflies with UTIV, TOSV, MVV, and Saddaguia virus (SADV) were 0.53% [21], 0.03% [19], 0.018% [22], and 0.57% [20,23], respectively. Similar results have also been reported in other countries of the Mediterranean basin. TOSV infection rates in sandflies of 0.22, 0.29, and 0.05% have been reported from Italy, France, and Spain, respectively [31,32,33]. In France, the infection rate of sandflies with the MASV was 0.37% [9].

The phylogenetic analysis indicated that PUNV, as it recognized nowadays as a new species [34], was isolated for three consecutive years, 2008, 2009, and 2010, in the site of Utique. During the entomological study carried out in 2009, PUNV was isolated from a pool of *P. perniciosus* females trapped in October, a period corresponding to the second main peak of sandflys’ activity. Compared with 2009, sandflies were not identified to the species level in 2010 because individual identification of specimens is time consuming; subsequently, this alternative approach revealed successful because three strains were isolated from the three PCR positive pools. Thus, PUNV was isolated from three pools of unidentified females trapped in October 2010. PUNV was isolated from sandflies of Northern Tunisia during three consecutive years, 2008, 2009 and 2010, supporting that it is endemic in this geographic area. Our findings suggest that PUNV is transmitted mainly by sandfly species of the subgenus *Larroussius* and particularly by *P. perniciosus*. The methodology used in this study did not allow for sandfly species identification of all PUNV positive pools, and did not eliminate the potential of multiple infecting viruses in sandflies, particularly viruses other than phleboviruses, such as insect-only viruses or other arboviruses. Although a 200-nt long sequence cannot confirm that the corresponding strains are identical to the original ones, this is sufficient to support that all PUNV strains that were detected from 2008 to 2010 belong to the same taxonomic entity, namely the Punique phlebovirus species. Complete genome analysis is desirable for fine-scale analysis of the microevolution of PUNV according to temporal and/or spatial criteria.

During the two entomological surveys carried out in 2009 and 2010, PUNV was detected in pools containing female sandflies. However, in 2008, PUNV was detected in both male and female sandflies as for TOSV [19,21]. Although experimentally demonstrated with viruses distinct from PUNV, the fact that sandfly-borne phlebovirus are infecting both male and female sandflies suggest that the virus circulates via several routes of transmission, that were confirmed experimentally, including trans-ovarial, venereal [35,36,37,38,39], and co-feeding on an infected sugar source [40].

The fact that sandflies enter into diapause as fourth instar larvae during winter and emerge as adults in spring [41,42,43] could explain the persistence of PUNV in nature and, subsequently, its endemicity, since it was isolated from sandflies collected in the same region for three consecutive years (2008, 2009, and 2010). However, the trans-ovarial and/or venereal transmission of PUNV has merit to be studied, as it has been with other phleboviruses [35,40]. The question of whether or not an amplifying host is necessary for virus maintenance and of the nature of this putative reservoir host also need to be investigated for PUNV. The fact that PUNV is endemic in Tunisia might provide a good model for further studies.

The question of the vertebrate reservoir remains unanswered. It was suggested that vertebrates play a minor potential role in the transmission and the ecology of the virus but rather provide the blood necessary for the development of eggs [44]. However, the question of a putative animal reservoir may be relevant as, for example, TOSV was isolated from the brain of a bat (*Pipistrellus kuhlii*) captured in an endemic area in Italy [45]. It is of major importance to point out that circulating phleboviruses were detected only during the second peak of sandfly activity, suggesting a buildup process of infection between sandflies and amplification hosts starting when adults emerge in the first peak of activity to reach a peak of infection late in autumn, in a similar way to *Leishmania* infection [25]. Taking into account that dogs are highly infected with PUNV or closely related antigenic variants compared with TOSV [46], more studies based on xenodiagnoses are needed to determine the potential role of dogs in the transmission of PUNV. Last, the fact that heathy dogs are not likely to play a role in the transmission of other phleboviruses raises the question whether *Leishmania*-infected dogs might be susceptible to phlebovirus, including PUNV infection.

Previous studies performed by our group showed that PUNV or closely related antigenic variants are able to infect humans, but at a low rate [47] compared with dogs [46]. The low sero-prevalence observed among humans compared with dogs could be explained by the zoophilic behavior of sandfly species of the subgenus *Larroussius,* mainly *P. perniciosus* [30]. More studies are needed to investigate the epidemiological impact of PUNV on humans and on dogs and the relationship with *Leishmania infantum*, an etiological agent of zoonotic visceral leishmaniasis [30,48,49], as both pathogens are transmitted by the same sandfly species belonging to the subgenus *Larroussius* [50].

## Figures and Tables

**Table 1:** Abundance of sandflies collected by CDC light traps in the Utique site in 2009.

Species	Sandflies Number		Total (%)
	Male	Female	
*P. perniciosus*	337	413	750 (85.91)
*P. longicuspis*	63	37	100 (11.45)
*P. perfiliewi*	5	5	10 (1.14)
*P. papatasi*	4	6	10 (1.14)
*S. minuta parotti*	3	0	3 (0.34)
Total	412	461	873 (100)

**Table 2:** Composition sandflies collected from Utique during 2009 according to date of collection, species, sex, and number (%) of specimens investigated for the presence of phlebovirus.

Date of Collection	Species	Numbers of Sandflies		
		Male	Female	
25 May 2009	*P. perniciosus*	90	17	(91.45)
	*P. longicuspis*	5	1	(5.12)
	*S. minuta parotti*	3	0	(2.56)
	*P. papatasi*	1	0	(0.85)
26 June 2009	*P. perniciosus*	20	15	(39.32)
	*P. longicuspis*	36	17	(59.55)
	*P. papatasi*	0	1	(1.12)
21 July 2009	*P. perniciosus*	13	30	(71.66)
	*P. longicuspis*	9	2	(18.33)
	*P. perfiliewi*	2	1	(5)
	*P. papatasi*	1	2	(5)
23 July 2009	*P. perniciosus*	30	30	(76.92)
	*P. longicuspis*	7	5	(15.38)
	*P. papatasi*	2	3	(6.41)
	*P. perfiliewi*	1	0	(1.28)
25 September 2009	*P. perniciosus*	90	150	(96.38)
	*P. longicuspis*	2	5	(2.81)
	*P. perfiliewi*	0	2	(0.80)
7 October 2009	*P. perniciosus*	94	171 *	(94.64)
	*P. longicuspis*	4	7	(3.92)
	*P. perfiliewi*	2	2	(1.42)

Abbreviation: * Detection of PUNV RNA.

## Data Availability

The data presented in this study are available on request from the corresponding author.
